# Perspectives on long-acting formulations of pre-exposure prophylaxis (PrEP) among men who have sex with men who are non-adherent to daily oral PrEP in the United States

**DOI:** 10.1186/s12889-023-16382-4

**Published:** 2023-08-28

**Authors:** Brooke G. Rogers, P. A. Chan, C. Sutten-Coats, A. Zanowick-Marr, R. R. Patel, L. Mena, W. C. Goedel, C. Chu, E. Silva, D. Galipeau, T. Arnold, C. Gomillia, K. Curoe, J. Villalobos, A. Underwood, C. Sosnowy, Amy S. Nunn

**Affiliations:** 1 Department of Medicine, Division of Infectious Diseases,, Warren Alpert Medical School of Brown University, Providence, 02903 USA; 2 Department of Psychiatry and Human Behavior, Warren Alpert Medical School of Brown University, Providence, 02903 USA; 3 Department of Social and Behavioral Sciences, Brown University School of Public Health, Rhode Island, Providence, 02903 USA; 4 Department of Infectious Diseases, Washington University School of Medicine, St. Louis, MO 63110 USA; 5 Department of Population Health Science, University of Mississippi Medical Center, Jackson, MS 39216 USA; 6 Department of Medicine, Division of Infectious Diseases, University of Mississippi Medical Center, Jackson, MS 39216 USA; 7 Department of Epidemiology, Brown University School of Public Health, Rhode Island, Providence, 02903 USA

**Keywords:** HIV prevention, Pre-exposure prophylaxis (PrEP), PrEP continuum, Men who have sex with men (MSM), Medication adherence

## Abstract

**Introduction:**

Pre-exposure prophylaxis (PrEP) persistence among men who have sex with men (MSM) in real world clinical settings for HIV prevention is suboptimal. New longer-acting formulations of PrEP are becoming available, including injectables, subdermal implants, and other oral medications. These longer-acting formulations have the potential to improve retention among those who have challenges remaining adherent to daily oral PrEP.

**Methods:**

We interviewed 49 MSM who had initiated but discontinued oral PrEP at three diverse clinics across the United States. We examined participants’ perspectives about long-acting PrEP formulations and how long-acting options could affect PrEP use using thematic analysis.

**Results:**

Participants were not very knowledgeable about long-acting formulations of PrEP but were open to learning about them and considering use. Participants were concerned about safety and efficacy of products given that they were still newer and/or in development. Finally, participants had clear preferences for oral pills, injectables, and then subdermal implants and were most interested in options that reduced the number of visits to the clinic.

**Conclusion:**

Long-acting formulations of PrEP are acceptable to MSM with suboptimal PrEP persistence and have the potential to improve PrEP persistence. However, many felt they needed more information on safety, efficacy, and use to consider these options. As these long-acting formulations are implemented, public health campaigns and clinical interventions to encourage may maximize uptake particularly among those who are not currently adherent to daily oral PrEP.

## Introduction

Once daily pre-exposure prophylaxis (PrEP) is a highly effective biomedical method for HIV prevention [1] and is recommended by the Centers for Disease Control and Prevention (CDC) for individuals at higher risk for HIV. HIV infection and incidence in the United States (US) disproportionately affects gay, bisexual, and other men who have sex with men (MSM) who represented 68% of new HIV diagnoses in 2020 [2]. Further racial and ethnic disparities exist. White MSM have a 1 in 11 lifetime risk of HIV, compared to 1 in 2 Black MSM and 1 in 5 for Hispanic/Latino MSM [2].

Uptake and retention in PrEP care among MSM have been suboptimal in real-world clinical settings [3–5]. PrEP efficacy is closely tied to adherence [6, 7] as there needs to be enough medication circulating in the body in order to provide adequate protection against HIV. In a study among MSM at three clinics in Providence, Rhode Island (RI), St. Louis, Missouri (MO), and Jackson, Mississippi (MS), 72% were retained in care at three months and 57% were retained in PrEP care at six months [8]. A San Francisco clinic serving MSM found an overall cumulative incidence of discontinuing PrEP at 13 months of 38% [9]. Among patients in a health system in Bronx, New York, retention at six months was 42% [10]. Prior studies have identified numerous factors that affect retention and adherence to daily, oral PrEP including having multiple anonymous sexual partners or having sex without using condoms [8, 11] low risk perception, [12] stigma, [3, 11, 13] disruptions in daily routine and substance use, [14] real and perceived side effects, [3, 11, 15, 16] cost, and lack of financial support [3, 11, 17].

One approach to overcome challenges associated with adherence to daily oral PrEP is long-acting PrEP products. Long-acting formulations of PrEP include long-acting injectables (LAI-PrEP), subdermal implants (SI-PrEP), and once monthly pills [18]. Recent clinical trials of LAI-PrEP, administered once every eight weeks, demonstrate equivalent protection to daily oral pills to prevent HIV and intramuscular cabotegravir (IM-CAB) was approved for use by the FDA in December 2021 based on two large randomized double-blind double-dummy studies in which IM-CAB was shown to have superior efficacy to daily oral TDF/FTC [19–21]. Despite being approved for use by the FDA, IM-CAB is still not included on most formularies, which means it is not listed as a potential medication to be prescribed by drug plans or insurance plans, which limits providers ability to prescribe the medication and its use in real-world clinical settings [18, 22]. SI-PrEP, similar to those used for contraception, provides sufficient drug concentrations to prevent HIV for up to one year [18]. Pill-based formulations containing antiretroviral drugs with longer half-lives than daily pills may last for a month [18, 22]. Thus, existing research suggests that long-acting formulations of PrEP are promising and may soon be available for use. In this study, we explored attitudes towards three forms of long-acting PrEP formulations currently in development, including LAI-PrEP, SI-PrEP, and monthly oral pills, among MSM in three US cities who had recently discontinued daily oral PrEP. As this group was both indicated for taking PrEP and was no longer taking oral PrEP it was hypothesized that this group would be potentially ideal for long-acting PrEP alternatives. The goal of the current study was to collect data on beliefs about long-acting PrEP options among individuals at higher risk for HIV acquisition who are suboptimally adherent to daily oral PrEP to inform real-world implementation strategies for this population.

## Methods

### Study participants and procedures

We interviewed 49 MSM across three PrEP clinics in the United States (19 in Providence, Rhode Island; 15 in Jackson, Mississippi; 15 in Saint Louis, Missouri). Participants for this study were recruited from a parent study that was an observational cohort study of MSM taking daily oral PrEP. Inclusion criteria for the parent study were assigned male at birth; identified as male; English or Spanish speaking; 18 years old or older; and reported sex with another male within the last 90 days; and, newly initiating or reinitiating PrEP. Participants were eligible and recruited for the current study if they were considered as having PrEP engagement or adherence difficulties defined as a missed a clinic visit (verified by medical records), had not picked up a prescription (verified by pharmacy records), or self-reported non-adherence to their PrEP medication for seven or more consecutive days on a follow-up survey. Research staff contacted individuals via phone up to three times to invite them to be interviewed. We sought balanced racial representation from all three study sites and purposefully sampled Black/African American and Hispanic/Latino MSM. We aimed for 10–15 interviews per site and stopped recruiting when we reached saturation. All participants self-reported not taking PrEP in the past 90 days. Participants received a $50 gift card for their participation in the qualitative interview. All study procedures were approved by the Institutional Review Boards at the three coordinating sites: The Miriam Hospital, Washington University in St. Louis, and University of Mississippi Medical Center.

### Data collection procedures

Interviews took place in-person in private rooms or online in Health Insurance Portability and Accountability Act (HIPAA)-secure online conferencing platforms. Interviews were audio recorded and transcribed by an external HIPAA-compliant transcription company and reviewed for accuracy by research staff. Researchers followed a semi-structured interview guide to conduct interviews. Individuals conducting the interviews wrote analytic memos following interviews to note contextual details and reflections following each interview. Notes were included as data to supplement qualitative analysis. Participants were asked about challenges with adherence to daily oral PrEP, intermittent PrEP, side effects and reluctance to take medication, missing clinical visits, insurance and copayments, laboratory tests, medical mistrust, other HIV prevention behaviors, and alternative forms of taking PrEP as part of a semi-structured interview. Interviewers described three formulations of long-acting formulations of PrEP: (1) a monthly oral pill, (2) subdermal implant PrEP, or (3) long-acting injectable PrEP and asked for participants’ opinions about each formulation including the pros and cons of each PrEP product and whether a long-acting form of PrEP would enhance their adherence. Responses to these questions and our interpretation follow below.

### Data analysis

We analyzed data using a thematic analysis approach [23]. First, we developed a preliminary coding scheme based on major topics of the study and questions in the interview guide. Each transcript was coded individually by at least one member of the research team. Five coders were involved in the coding process. Coders completed four transcripts at a time and met twice monthly with other coders on the research team to compare results, resolve discrepancies, and refine the coding scheme. During analysis, we used a general inductive approach to identify emergent codes, themes, patterns and conclusions from the interviews [24]. Quality checks were conducted on 20% of all transcripts and interviewer notes via iterative coding by at least two coders. A third, senior coder, then reviewed these transcripts to confirm that transcripts had been coded to agreement. Coded transcripts were then reviewed by the first author, summarized, and themes were constructed in an iterative process with assistance from other study staff members.

## Results

### Participant characteristics

Across all sites, the average age was 29.5 years. The majority were White (n = 36; 73.5%), Non-Hispanic/Latino (n = 44; 89.8%), and all identified as sexual minority men, which is a term that is inclusive of gay, bisexual, and/or queer identified men. Most had private health insurance (n = 36; 73.5%). The sample was diverse with regards to years of education, employment, and salary. Prior to enrollment in the study, most had no prior experience with taking PrEP (n = 37; 75.5%) Additional demographic information is described in Table 1.

**Table 1:** Demographic and behavioral factors among participants of non-adherent PrEP interviews

	Mississippi		Missouri		Rhode Island		Total	
**Age (years) (median, IQR)**	(24.0, 22.5–26.0)		(28.0, 22.0-30.5)		(32.9, 19–69)		(29.4, 23.0-43.4)	
	N = 15	%	N = 15	%	N = 19	%	N = 49	%
**Race**								
White	1	(6.7%)	10	(66.7%)	12	(63.2%)	23	(46.9%)
Black	14	(93.3%)	3	(20.0%)	1	(5.3%)	18	(36.7%)
Asian	0	(0%)	1	(6.7%)	1	(5.3%)	2	(4.1%)
Other	0	(0%)	0	(0%)	1	(5.3%)	1	(2.0%)
Multiple Races	0	(0%)	1	(6.7%	2	(10.5%)	3	(6.1%)
Decline to Answer	0	(0%)	0	(0%)	2	(10.5%)	2	(4.1%)
**Ethnicity**								
Hispanic/Latino	0	(0%)	0	(0%)	4	(21.0%)	4	(8.2%)
Non-Hispanic/Latino	15	(100.0%)	15	(100.0%)	15	(78.9%)	45	(91.8%)
**Sexual Orientation**								
Gay	7	(46.7%)	11	(73.3%)	15	(78.9%)	33	(67.3%)
Bisexual	8	(53.3%)	4	(26.7%)	1	(5.3%)	13	(26.5%)
Queer	0	(0%)	0	(0%)	3	(15.8%)	3	(6.1%)
**Health Insurance**								
Private	5	(12.2%)	15	(100%)	14	(84.2%)	34	(69.4%)
Medicare	0	(0.0%)	0	(0%)	1	(5.3%)	1	(2.0%)
Medicaid	4	(26.7%)	0	(0%)	4	(21.1%)	8	(16.3%)
None	6	(40.0%)	0	(0%)	0	(0%)	6	(12.2%)
**Education Level**								
Some high school	1	(6.7%)	0	(0%)	1	(5.3%)	2	(4.1%)
High school graduate	5	(33.3%)	1	(6.7%)	1	(5.3%)	7	(14.3%)
Some college/technical school	7	(46.7%)	4	(26.7%)	4	(21.1%)	15	(30.6%)
College graduate	2	(13.3%)	8	(53.3%)	8	(42.1%)	18	(36.7%)
Graduate School	0	(0%)	2	(13.3%)	5	(26.3%)	7	(14.3%)
**Employment Status**								
Full-time	6	(40.0%)	10	(66.7%)	14	(73.7%)	30	(61.2%)
Part-time	5	(33.3%)	2	(13.3%)	3	(15.8%)	10	(20.4%)
Unemployed	1	(6.7%)	0	(0%)	1	(5.3%)	2	(4.1%)
Student	2	(13.3%)	3	(20.0%)	0	(0%)	5	(10.2%)
Retired	0	(0%)	0	(0%)	1	(5.3%)	1	(2.0%)
Other	1	(6.7%)	0	(0%)	0	(0%)	1	(2.0%)
**Annual Income**								
Less than $10,000	3	(20.0%)	2	(13.3%)	2	(10.5%)	7	(14.3%)
$10,001 to $20,000	3	(20.0%)	2	(13.3%)	3	(15.8%)	8	(16.3%)
$20,001 to $50,000	5	(33.3%)	6	(40.0%)	7	(36.9%)	18	(36.7%)
$50,001 to $75,000	2	(13.3%)	2	(13.3%)	3	(15.8%)	7	(14.3%)
$75,001 or more	1	(6.7%)	3	(20.0%)	4	(21.1%)	8	(16.3%)
Declined to answer	1	(6.7%)	0	(0.0%)	0	(0.0%)	1	(2.0%)
**Ever taken PrEP prior to study**								
Yes	6	(40.0%)	3	(20.0%)	3	(15.8%)	12	(24.5%)
No	9	(60.0%)	12	(80.0%)	16	(84.2%)	37	(75.5%)

### Main findings

Overall, participants had little to no prior information about long-acting formulations of PrEP which led them to express (1) a desire for more information around use of these products as well as safety and efficacy data and (2) preferences for these formulations based on their existing knowledge and understanding of how they might work in practice. We explore these two themes below with illustrative quotes from participants. There were no notable differences by site or by other demographic characteristics.

### Lack of knowledge and need for more safety and efficacy data for long-acting formulations

Participants were generally unaware of long-acting formulations of PrEP that were undergoing research, testing, and development at the time interviews were conducted. Interviewers often had to introduce the multiple forms of long-acting formulations and answer several questions on how they were delivered, what medications were involved, and potential reported side effects from ongoing trials to date. Participants expressed concerns about newer formulations because of a lack of information and were interested in learning more so they could better evaluate whether long-acting formulations were safe and as effective as once daily oral PrEP. Participants were interested in having more data from clinical trials research to help make their decision but generally liked the idea of there being alternative options for PrEP:

“I don’t know because in my opinion, I know with clinical trials and everything, I would rather take a pill a day. If that [long-acting PrEP] was an option for us, that would be very interesting…not worrying about taking a pill every day and everything.” – (20 years old, Black/ African American, Missouri)

“I’m one that believes in science and backs science, so if the studies were there and the research was there...I would much rather do that than have to take a pill every day. I love the idea of it.” - (34 years old, Black/ African American, Missouri)

Participants were motivated to learn more about long-acting formulations and shared that they would have to do more personal research on the topic in order to evaluate whether it was good fit:

“I’ve never had any experience with it. I think that’d be something that I’d have to do more research on…but I think that’d something I could be interested in if it’s only once a year. Then that’s really, really easy then in terms of maintaining and all of that stuff...” – (24 years old, White, Mississippi)

Participants were not interested in being the first to try new formulations for fear of being “test subjects” on a new medication. Instead, they preferred waiting until several individuals were taking these PrEP options to evaluate safety and efficacy:

“I would say—I would not want to be one of the first people to do it. I would want to wait a few years until there have been major studies about it. I wouldn’t be volunteering to test that one out.” (37 years old, White, Rhode Island)

When it came to a monthly oral pill some were concerned about being able to remember for just one day a month and others were concerned that the efficacy would be lessened over the time:

“…I would be really skeptical of the research first that proved to me that a pill on the 1st was still working with the same levels on the 30th as it was on the 1st. As long as that research was there and could prove that my antiviral levels were the same on the 30th as they were on the 1st, then, yeah, I would love that, but I would be very skeptical at first.” - (32 years old, White, Missouri)

### Preferences and considerations for long-acting formulations

Participants were open to considering novel PrEP formulations and described taking daily oral medication to be a challenge that long-acting medications could address, particularly, if it meant fewer clinic visits. The strongest preference was for once monthly oral medication, followed by long-acting injectable PrEP, and then subdermal implants. Participants thought that long-acting options would be most convenient if they could be provided in local medical offices and even pharmacies rather than specialized clinics and/or combined with PrEP care visits at the clinic. Many acknowledged that long-acting options might reduce clinic visits, which would be more likely to retain them in care:

“I’d rather have my appointment once a month or once every couple months just like I have my appointment once every three months with the doctor, than have to take a pill every morning. I think that those would make it more accessible for anyone who’s capable of going in person to the hospital.” (22 years old, White, Missouri)

Although there was inter-individual variation, in general, participants showed the most interest for a monthly pill, moderate interest for an injectable version, and least interest for subdermal implants. The monthly pill was palatable because it was a modification on the existing way in which a pill is taken. Individuals saw a once monthly pill as closest to a daily, oral pill, and therefore expressed a higher level of comfort with this option:

“I would do the once-a-month pill first. I feel like that’s the most viable option for me, just ’cause I feel like taking a pill is easy. Second would be the injection. Again, that’s just ’cause I feel like it’s more of a convenience thing of getting the shots. It’s a little inconvenient to have to go every two months to get it or every—then last would be the implant.” – (34 years old, White, Rhode Island)

When discussing subdermal implants, participants were concerned about there being something introduced to their body somewhat permanently. There were questions about the material in the implant, how it would be inserted and/or removed, and what would happen once it was introduced to a body:

“I would probably just want—I’d probably just stay with the pill because I’m not sure what we’re implanting… I don’t know what’s going inside of me. Is it a piece of metal? I think, me, I’d be leery about that. To me, the first two, I’d rather have—between injection, implant, and pill, I’d rather have the pill once a day or once a month.– (63 years old, White, Rhode Island)

Some individuals expressed concern that an implant could create other problems including with travel and the potential for setting off metal detectors or other screening devices: “I’m not big on having additional devices in. Considering the fact, also, you have a lot of people who travel. Say if that device does trigger that metal detector or so—the next thing you know, they don’t have a way to prove what is in you. You have TSA patting you down. They’re wondering why are you going off like a metal detector? What do you have inside you?”– (27 years old, White, Missouri)

Participants expressed concerns about bodily autonomy and having “foreign objects” implanted in their bodies:

“I don’t want a foreign object in my body. I don’t like the whole implant thing.” (26 years old, African American/Black, Mississippi)

There were also concerns about the seeming permanency of a subdermal implant compared to a medication. Although implants are removable, many participants thought they were permanent and would be with them forever:

“I’m not comfortable with getting something permanently put under my skin.” (30 years old, multiple race, Missouri)

There were many questions as to how subdermal implants would work, and overall, individuals experienced more hesitancy and were concerned about the safety and efficacy of this option:

“There’s so many variables I don’t know, like how is it implanted? How much is in there? What happens if it releases it too quickly? I’d have a lot of questions to decide whether or not I’d be okay with it.” (29 years old, White, Rhode Island)

Overall, injections or “long-acting injectable” PrEP were more positively received than subdermal implants. One noted advantage of injectable PrEP was the lack of “evidence” of taking PrEP and it being a more discrete means of taking the medication to prevent HIV, and thus beneficial for individuals who were concerned about disclosing their PrEP use:

“I very much like that idea, especially for people, like, concerned about, you know, like the stigma of, like, a big blue Truvada pill. .. or like, families being aware and stuff. That’s a pretty easy way to, like, not have anyone ever know about that.” (24 years old, African American/Black, Mississippi)

Participants suggested that long-acting injectable PrEP options would be most beneficial if they were able to coordinate their regular PrEP clinic visits and lab reviews at the same day and time that they were receiving the injections:

“Okay. I would be okay with that. It would be perfect if they line it up with when you come and get your blood tested, and then you do the injection at the same time. If you make it convenient and combine it with other procedures that has to be done, then I would be okay with it.” – (26 years old, Asian, Missouri)

However, getting to the office for additional scheduled visits for the injections was considered a burden and a potential barrier to staying engaged in PrEP care:

“It depends on where I’d be able to get the injectable. If I could just go to my pharmacy and get it done, I would probably do that over the pill, or the daily pill, at least. If they do the monthly pill, and they give you, I don’t know, a couple of months’ supply at a time, I would probably do that as well, just because the office, or the clinic is a little far away from me, so having to visit the clinic every month, like once a month, would be a little much.” – (24 years old, White, Missouri)

There were also concerns about missed appointments and how that would affect adherence and efficacy. Individuals noted that with daily oral medication, if you miss an appointment you can continue to take PrEP and/or have a prescription filled. However, if relying on the visit in order to receive the PrEP medication there may be days or even weeks without medication. Individuals were interested in whether they could mix and match options (for example, take oral medication to bridge the time until their next injectable visit) to help optimize PrEP coverage:

“I like that idea to be honest. The only thing that worries me is if I can’t get into the doctor to get the new shot, do you—do they know if—like I’m taking the medication now, the pill. If I weren’t being able to get in, would I be able to take the pill in the meantime until I can get into the office or is that not known yet?” – (25 years old, White, Missouri)

Overall, participants were not knowledgeable about other forms of PrEP at the time this research was conducted. They were interested in learning about alternative, long-acting forms of PrEP. Most had some concerns about trying new forms of PrEP and were interested in seeing more data on the efficacy and safety of these newer formulations. Additionally, most participants preferred oral PrEP and several others were open to injectable PrEP while fewer were interested in subdermal implants.

## Discussion

This study was among the first to evaluate the perspectives of MSM who had initiated and discontinued oral PrEP to examine how long-acting formulations of PrEP including LAI-PrEP might impact retention and adherence for those who have problems with daily adherence to oral PrEP. Challenges to daily oral PrEP and/or suboptimal retention in PrEP are multifaceted and include transportation and out-of-pocket costs, misinformation on media and in personal networks, frequency and timing of appointments, and, changes in sexual behavior and low perceived risk for HIV.

Similar to existing studies, PrEP knowledge continues to be a barrier to willingness to use PrEP [25]. Because most participants had not previously heard of long-acting formulations, they felt that there were many unknowns and that they would need to know more and feel confident in the efficacy and safety before considering uptake. Being informed and reassured about product safety would likely lessen some of these concerns. Information campaigns, educational materials, and providers should address those concerns and be clear about product safety and potential risks.

Although most study participants were not familiar with long-acting formulations of PrEP, they were interested and willing to consider at least one formulation. Participants favored the convenience of being able to take PrEP less frequently and remain consistent with their PrEP use when other things fluctuated, such as their schedule or sexual activity. For all three formulations, participants were concerned about potential short- and long-term side effects, safety, and efficacy. Participants were comfortable with longer-acting (once monthly) oral medication because daily oral medication has already been approved for use for almost a decade, they were the least informed and most concerned about subdermal implants, and were moderately comfortable with long-acting injectable forms and thought it would be convenient if offered in multiple care locations including primary care, pharmacy-based walk-in clinics, etc. (not just infectious disease and sexual health clinics) and/or combined with other medical appointments.

### Monthly oral medication

At the time we were conducting interviews, Phase II and Phase III clinical trials for once monthly oral islatravir were ongoing. However, as of December 2021, Merck paused the IMPOWER 22 (for cisgender women at high-risk for HIV) and IMPOWER 24 trials (for cisgender men who have sex with men and transgender women at high-risk for HIV) due to safety concerns with changes in CD4 + T cells [26]. The future of islatravir and other long-acting oral formulations remains unclear.

### Subdermal implants

Fewer studies have examined SI-PrEP, but also demonstrate preliminary evidence for acceptability with some concerns around insertion and removal [27–29]. At the time we started this study, there were several trials of SI-PrEP of islatravir that were ongoing. However, as of December 2021, these trials have been halted by FDA due to observations in decreases in total lymphocyte and CD4 + T cells [30]. With FDA’s clinical hold, no new studies may be initiated and individuals who were enrolled in the trials will no longer receive the study drug. SI-PrEP is still worth considering in future trials given the relative ease and low patient burden for adherence and persistence.

### Long acting injectables

Participants expressed a preference for injectables if the schedule aligned with current clinical guidelines for appointments; however, as currently available it requires bimonthly appointments. While LAI-PrEP would decrease the daily burden of taking a pill, the clinical burden would increase by requiring clinic visits every two months. The frequency of injections and clinic visits may make it a less desirable option for some [31]; however, for others, the benefits of LAI-PrEP may override concerns of medical visits because it can be a discrete form of protection and does not require taking a daily oral medication [27, 31–33].

Gaining a better understanding of how these factors are considered by potential users can help plan for future implementation and scale-up of LAI-PrEP. Since conducting this study, intramuscular cabotegravir (IM-CAB) has been approved for use by the FDA as the first LAI-PrEP by the FDA to be taken as two initial intramuscular injections given 1 month apart and then administered every 2 months afterwards. This is the first non-oral PrEP therapy to become available and in clinical trials it showed superior efficacy to daily oral TDF/FTC [19–21] As of December 20, 2021 IM-CAB has been approved for use. If scaled up, it is important to consider the impact that IM-CAB as LAI-PrEP could have on HIV prevention given that it would increase coverage for individuals who have difficulty with daily adherence to an oral medication [32].

### Real world implementation and health equity

While long-acting formulations have been seen as highly acceptable among patient populations [34] and show promise for expanding options and addressing some barriers to PrEP adherence and persistence, there are “real world” concerns about LAI-PrEP to adequately address the needs of the population who could benefit. Cost for generic TDF/FTC daily oral PrEP is $1/day (or $30/month) while LAI-PrEP is approximately $3000/injection [35–37]. For expansion of the use of LAI-PrEP, insurers would need to add it to their formulary. This may be particularly important for state-based and federally based government providers (e.g., Medicaid, Medicare) since they provide safety net care for individuals without private insurance [38]. However, the difference in price might make it a less desirable option for insurers and patients alike. Additionally, LAI-PrEP may be less desirable because it requires more frequent visits to clinical sites than oral PrEP. Current clinical guidelines for LAI-PrEP include injections given one month apart for two consecutive months followed by a single injection every two months afterwards during a clinical visit [40].

Increased clinic visits may present a significant barrier for individuals especially those who have difficulty with attending clinic appointments because of transportation or work schedules. Additional clinic appointments also may mean additional co-pays or charges for visit attendances, which could be a financial barrier for some. Finally, concerns about lack of information about long-acting options, short- and long-term side effects, safety, and efficacy may be addressed through clinical care, but these concerns may deter others from considering PrEP or seeking care. Issues such as awareness, [3, 39–41] knowledge, [42, 43] and access to PrEP [44, 45]; affording PrEP; [46] medical mistrust; [47–49] stigma [13, 16, 47, 50] and changing cultural attitudes [11, 51] also need to be addressed to optimize uptake of any form of PrEP. Nevertheless, long-acting formulations may be an important next step to improving PrEP uptake, retention, and persistence.

### Limitations

Our study strengths must be interpreted with consideration of its limitations. This sample was recruited from within clinical care settings and thus reflects those individuals who have been able to access, uptake, and maintain PrEP care. This group of individuals is systematically different from those who are not able to access care. Additionally, this study was conducted as part of a larger study and is therefore reflective of a group of individuals willing to participate in research, which may make them somewhat different than those who access care and are unwilling to participate in research [52]. Future research should seek to increase the diversity of those participating in these studies to identify and address unique challenges to retention in care among those who are both at highest risk and traditionally underrepresented in research trials.

## Conclusion

This study is unique in that it examines preferences for long-acting PrEP products among a group of individuals who have used daily, oral PrEP and had adherence issues, which might be the population for whom long-acting formulations of PrEP are most beneficial. Our study findings provide insight on factors that are important to consider for promoting uptake prior to the scaling up and out of long acting formulations of PrEP. This study represents the perspectives of sexual minority men who initiated and discontinued daily oral PrEP in diverse clinical settings across three U.S. cities and offers important information about preferences for long-acting PrEP formulations. Real world implementation of daily oral PrEP has been a challenge and clinicians have sought ways to increase uptake and improve retention. Long-acting formulations of PrEP, including LAI-PrEP, SI-PrEP, and once monthly oral pills hold promise for improving PrEP utilization among higher incidence groups specifically because they address challenges in daily adherence and stigma.

## Data Availability

The datasets generated and/or analysed during the current study are not publicly available but are available from the corresponding author upon reasonable request.
